# Medicinal Plants and Isolated Molecules Demonstrating Immunomodulation Activity as Potential Alternative Therapies for Viral Diseases Including COVID-19

**DOI:** 10.3389/fimmu.2021.637553

**Published:** 2021-05-13

**Authors:** Hassan A. Alhazmi, Asim Najmi, Sadique A. Javed, Shahnaz Sultana, Mohammed Al Bratty, Hafiz A. Makeen, Abdulkarim M. Meraya, Waquar Ahsan, Syam Mohan, Manal M. E. Taha, Asaad Khalid

**Affiliations:** ^1^ Department of Pharmaceutical Chemistry, College of Pharmacy, Jazan University, Jazan, Saudi Arabia; ^2^ Substance Abuse and Toxicology Research Centre, Jazan University, Jazan, Saudi Arabia; ^3^ Department of Pharmacognosy, College of Pharmacy, Jazan University, Jazan, Saudi Arabia; ^4^ Department of Clinical Pharmacy, College of Pharmacy, Jazan University, Jazan, Saudi Arabia

**Keywords:** COVID-19, phytoconstituents, immunomodulation, cellular immunity, humoral, viral infections, traditional Chinese medicine

## Abstract

Plants have been extensively studied since ancient times and numerous important chemical constituents with tremendous therapeutic potential are identified. Attacks of microorganisms including viruses and bacteria can be counteracted with an efficient immune system and therefore, stimulation of body’s defense mechanism against infections has been proven to be an effective approach. Polysaccharides, terpenoids, flavonoids, alkaloids, glycosides, and lactones are the important phytochemicals, reported to be primarily responsible for immunomodulation activity of the plants. These phytochemicals may act as lead molecules for the development of safe and effective immunomodulators as potential remedies for the prevention and cure of viral diseases. Natural products are known to primarily modulate the immune system in nonspecific ways. A number of plant-based principles have been identified and isolated with potential immunomodulation activity which justify their use in traditional folklore medicine and can form the basis of further specified research. The aim of the current review is to describe and highlight the immunomodulation potential of certain plants along with their bioactive chemical constituents. Relevant literatures of recent years were searched from commonly employed scientific databases on the basis of their ethnopharmacological use. Most of the plants displaying considerable immunomodulation activity are summarized along with their possible mechanisms. These discussions shall hopefully elicit the attention of researchers and encourage further studies on these plant-based immunomodulation products as potential therapy for the management of infectious diseases, including viral ones such as COVID-19.

## Introduction

Severe acute respiratory syndrome coronavirus-2 (SARS-CoV-2) is a novel strain of coronavirus first isolated from humans in December 2019 in Wuhan city, China. It is highly contagious and produces a disease known as COVID-19, which has pneumonia-like symptoms and has resulted in a pandemic. COVID-19 can infect all age groups, showing relatively severe symptoms in people over 60 years of age (1) and currently no medications are approved for its therapy. For a disease without treatment, preventive medicines are prescribed and immunomodulators with antivirals can serve the purpose. To control such infections, vaccination is considered to be an effective method; however, it is known to prevent the incidence of acute respiratory infections in healthy individuals only to a certain extent (2). As seen in all viral infections including SARS-CoV-2, the virus-specific T cells responsible for cell-mediated immunity and B lymphocytes for humoral immunity, play important roles in the adaptive immune response by the body. There is an increase in neutrophils, interleukin (IL)-6 and C-reactive protein and a decrease in the total number of lymphocytes (3). The activation of T lymphocytes plays a role in exacerbation of inflammatory responses, whereas the B lymphocytes help in producing specific neutralizing antibodies for the virus (4, 5).

Herbal products are recognized as complementary approach to modern medication; consequently, immunomodulation agents from natural sources have proven to be safe and effective alternatives (6, 7). Immunomodulators stimulate the body’s natural defense against pathogens including viruses, thereby maintaining immune-system homeostasis and could be an effective way to prevent viral infections. Malfunctions or imbalances in the immune system are associated with a range of chronic diseases, including allergies, cancer, inflammatory bowel diseases, autoimmune disorders, viral infections, and many others. Carotenoids, terpenoids, flavonoids, polyphenols (e.g., stilbene derivatives), organosulfur-containing compounds (e.g., allyl isothiocyanate, hydrocinnamic acid derivatives) and polysaccharides are important phytocompounds with known chemical structures and considerable immunomodulating activities (8).

Among other popular therapeutic approaches, immunotherapy has been utilized indirectly for the treatment of cancer. Recently, immunotherapy demonstrated positive responses in cancer treatment with clinical successes by blocking antibodies at two immune checkpoints, namely, cytotoxic T-lymphocyte-associated protein 4 and planned cell death protein 1, in addition to chimeric antigen receptor-transduced T cells (9). Globally, around 180 million people are chronically infected with hepatitis C virus and owing to the quasispecies nature of the virus, the development of effective vaccination has long been a challenging task (10). Cerebral malaria (CM), often occurs in immunocompromised individuals along with the failure of antimalaria treatment. Plant-based natural products such as curcumin have demonstrated promising immunomodulation activity and can be used as an adjunctive therapy for CM (11). Dengue is another mosquito-borne viral disease causing high levels of pro-inflammatory cytokine production, leading to endothelial activation and vascular leakage. Antioxidants and natural immunomodulating agents from plant sources have demonstrated promising results in the treatment of a number of viral infections (12).

Dietary polysaccharides are the prebiotics which are known to affect gut microbial ecology. There has been a direct correlation between nutrients present in the diet and the immune function (13). A number of micronutrients, macronutrients and the gut microbiome mediate the immunological effects of body. The microbial transformation of dietary components to its metabolites has a considerable effect on the physiological and immunological processes. There is a close relationship between the bacterial metabolism and the efficacy of phytochemicals as it may increase or decrease upon metabolism (14). Following the advancements made in the field of microbial ecology, immunology and metabolomics, the impact of gut microbiota on host nutrition, immune modulation, resistance to pathogens, intestinal epithelial development and activity and energy metabolism has been recognized (15). Plant-based nutritional foods are known to improve the population of beneficial bacteria present in intestines which help and constitute up to 85% of the immune system (16). Spices used in the food and their secondary metabolites (17) also showed the immunostimulation potential and it was observed that the COVID-19 cases were more prevalent in countries with lesser gram spice supply per capita per day (18). Non-digestible nutrients that selectively promote the growth of normal intestinal bacterial flora are known to have beneficial effects on the large intestinal lining. After being hydrolyzed by gut bacteria, these nutrients may either bind to epithelia or get absorbed in the systemic circulation to stimulate the immune system (19, 20). Among studied immunomodulation factors (including recombinant interferon-gamma (rINF-γ), interferon-alpha (rINF-α), ampligen, and CL246), a total of 738 factors exhibited significant antiviral activity and were recommended for prophylactic or therapeutic use in early-stage infections of several species of viruses (21).

The aim of this review was to summarize the most important medicinal plants which displayed good immunomodulation activity from the recently published literature. It also covers the possible mechanism of immunomodulation for these plants and the role of natural products in the treatment and management of viral infections. Recently published articles from commonly used search engines such as Pubmed, ScienceDirect, Google Scholar, Microsoft Academic Research, etc. which were related to the scope of the review were included. A good number of reviews are present discussing various plants and their bioactive constituents demonstrating immunomodulation and antiviral activities, however, an updated review focusing on the immunomodulation activities of important medicinal plants and their isolated molecules along with their mechanisms is still warranted.

## Immune System Responses to Viral Infection

Infection by pathogenic viruses causes a number of infectious diseases, such as influenza, smallpox, human immunodeficiency virus (HIV)/AIDS, measles, and newly identified COVID-19 (22). The body’s defense system recognizes the infecting organisms such as viruses and bacteria as foreign particles through innate or adaptive immunity systems. In case of innate immunity, foreign microbes are identified by pattern-recognition receptors (PRRs) situated on the surface of innate immune cells. Subsequently, the invading organisms are destroyed by certain defense processes, such as cytotoxin generation by natural killer (NK) cells and macrophage-mediated phagocytosis (23, 24). In adaptive immunity, microorganisms are recognized by T and B lymphocytes through cell surface-antigen-specific receptors (T cell and B cell receptors) and the invaders are eliminated through cytotoxic responses (e.g., neutrophils, CD45+ lymphocytes, CD8+ T cell, monocytes, and mature B lymphocytes) and antibody production (23, 25). However, pathogenic microbes often succeed in infecting the biological system despite the strong defense mechanisms even in immunocompetent individuals because of a number of immune-evasion tactics, including surpassing recognition by immune cells and immobilizing immune responses (26–28).

Owing to the high sequence variability of viruses, they can avoid immune responses *via* the variation and interruption of antigen generation from virus-infected cells (29, 30). Viruses can interfere with the antiviral signaling of the immune system by altering genetic materials, reducing cytokine generation (31), and hindering the expression of PRRs and their adapter (32). Viruses can also affect the immune response by reducing the cytotoxic function of T cells (33) and reducing NKG2D and MHC-1 molecules, thereby suppressing immune stimulation (34, 35). For example, dengue and West Nile viruses escape immune response through the blockage of IFN-α/IFN-β receptors (36), and influenza viruses achieve gene mutations involved in antigen binding through “antigen drift” (37). In another way of escaping immune responses, virus particles enter a host cell in the dormant state and subsequently are reactivated when the cell immunity weakens. Consequently, the detection of viruses becomes difficult because the infected cells start upregulating the expression of latency-related genes rather than lytic viral genes (38).

These viruses can be best exemplified by human papillomavirus (HPV), which can adopt a dormant state and after assuming dormancy, the migrating HIV-infected cells can be reactivated by differential responses to drugs (39, 40). These viruses can create a favorable environment for infection through alteration of various metabolic processes of host cells; such as enhanced fatty acid synthesis, change in aerobic glycolysis, and glutaminolysis (41, 42). Additionally, enhanced lactic acid production creates an acidic microenvironment that helps in virus pathogenesis as the fusion of an enveloped virus to the host cell membrane occurs more effectively at acidic pH (25, 43). For instance, Zika virus, hepatitis B virus (HBV), and HIV induce the dysregulation of glycolytic processes through enhanced glucose influx, lactic acid production, and GLUT1 expression in host cells (44). The short-term exposure of Ebola virus to an acidic environment may result in increased fusion rate to the host cell membrane (45).

## Mechanism for Innate Antiviral Immunity

Innate immunity are the non-specific immunity which includes expression of factors resulting into inhibition of virus particles and slowing down the infections in a rapid manner without adaptation or generation of long-lasting memory for protection. The innate antiviral responses are critical in order to mobilize the protective immunity. Upon viral infection, the innate immunity cells get activated mainly through the germline-coded pattern recognition receptors (PRRs) which are present either on the cellular surfaces or within distinct intracellular compartments. The PRRs include the family of Toll-like receptors (TLRs), the retinoic acid-inducible gene-I-like receptors (RLRs), cytosolic DNA sensors and the nucleotide-oligomerization domain-like receptors (NLRs) (46). These receptors are mainly triggered by the exposure of viral nucleic acids and proteins. These PRRs present in a wide array of cellular components sense the presence of viral antigen and quickly respond to the viral infection and replication of these viruses in the cellular compartments.

Activation of family of TLRs including TLR-3, 7, 8 and 9 mediates the production of type-I interferons (IFN-Is) and pro-inflammatory cytokines. The cytokines are responsible for the production of a number of inflammatory mediators causing inflammation, fever and pain; whereas, the IFN-Is are the important mediators of antiviral immunity and are responsible for the antiviral activities (47). Similarly, RLRs sense and detect the viral RNA in the cytosol resulting into the induction of IFN-Is. The RLRs can generally differentiate between the viral RNA and the host RNA owing to the distinct features present in the viral RNA (48). The RLRs can detect the double-stranded viral RNA and activate NF-κB and interferon regulatory factors (IRFs) through the adaptive protein, mitochondrial antiviral signaling protein (MAVS), a protein that resides in the mitochondrial membrane and orchestrates host antiviral innate immune responses to the RNA-virus infection (49). Another important mechanism by which the host defense system neutralizes and kills the pathogens is *via* expression of natural antibodies (NAbs), which are innate-like serum antibodies (50). These NAbs play important role in early immune response against the viral infections and are crucial part of the host defense.

The activation of PRRs initiates signal transduction *via* a series of adaptor proteins leading to the expression of a number of transcription factors such as NF-κB, IRF-3, IRF-7 and several kinases; consequently the production of antiviral IFN-Is. However, certain viruses have evolved in such a way that they can block the innate immune-signaling and hinder the antiviral responses by host. They escape detection by PRRs, can block the activation of adaptor proteins and kinases and can disrupt the transcription factors; thereby successfully invade the host cells (51). Various components of innate immunity include macrophages/monocytes, neutrophils, granulocytes, natural killer (NK) cells and dendritic cells. These cells can identify and kill the viral pathogens by phagocytosis, cleavage and by cytotoxic activity (52).

## Treatment of Viral Diseases Using Natural Immunomodulators

The treatment of infectious diseases involves either directly killing the infecting microbes or therapeutically modulating the body’s immune responses to eradicate pathogenic organisms from the body without causing injury to the host cells. The immunity to microbial attacks might involve nonspecific responses (innate immunity) to common antigens or acquired responses (adaptive immunity) to specific microbes (53, 54). Multiple potential targets have been identified to modulate the immune responses of body for the microbial attacks. Considering that viruses are obligate intracellular pathogens and entirely dependent on the host, direct therapy using pharmaceuticals for viral infection is difficult (55). The development of vaccine against a particular viral infection is a slow and tedious process and targeting immune responses becomes important for the intervention of these diseases (56). In recent years, immunotherapy has been developed as a promising therapy in form of complementary treatment for the diseases caused by viral infections (56–59).

**Figure 1:**
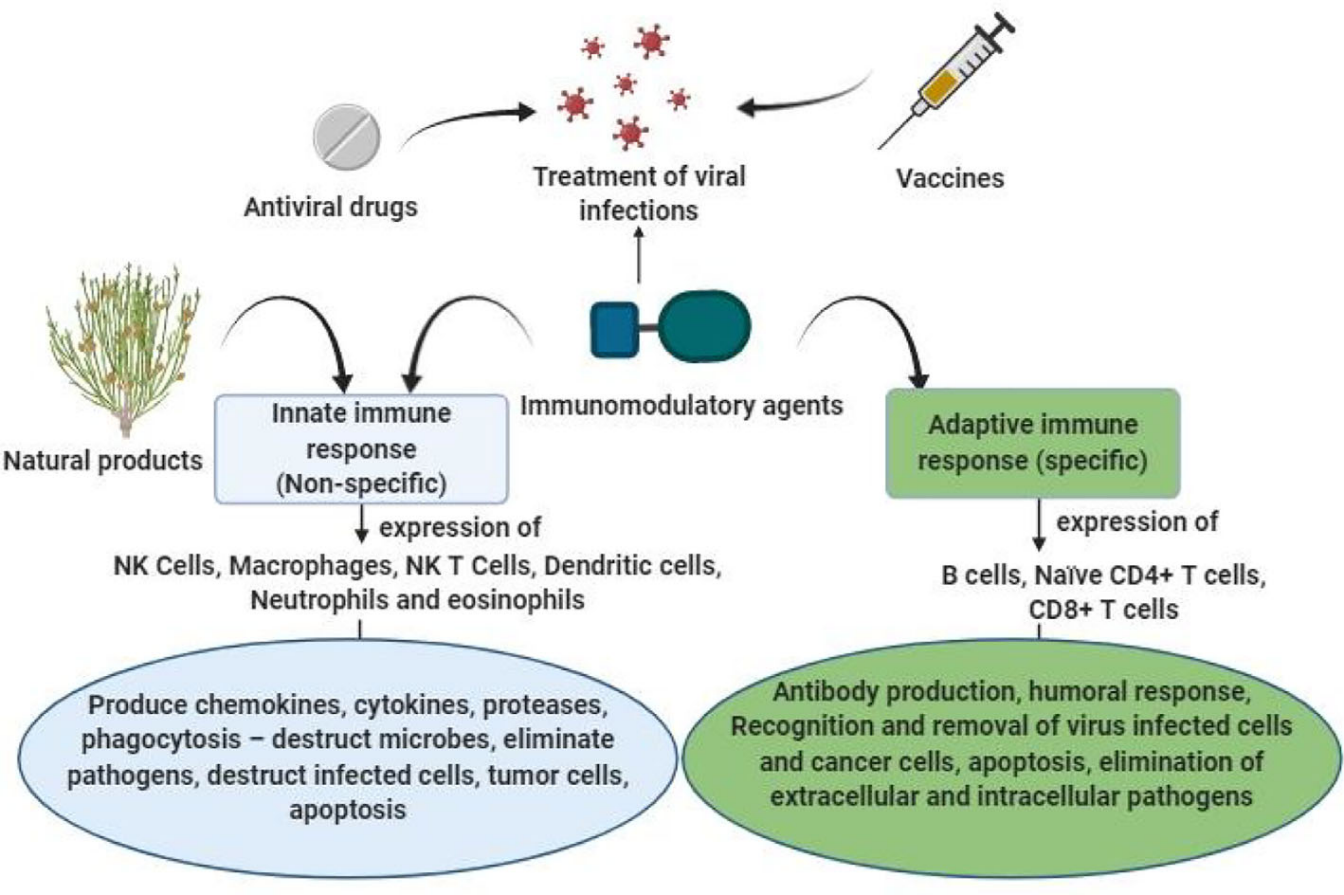
Possible role of natural immunomodulators for prevention and treatment of viral infections.

Natural products are known to primarily stimulate the nonspecific immune responses (innate immunity), in which the mediators of immune system including cytokines, macrophages, neutrophils, acute-phase proteins and monocytes provide instant defense against pathogens (60). Pathogens express different factors known as pathogen-associated molecular patterns (PAMPs), which are recognized by the host to perceive the presence of pathogens. PAMPs are recognized by host sensors using PRRs triggering a sequence of immune reactions through the generation of type-1 IFNs, cytokines, and chemokines. PRR families include NOD-like receptors, Toll-like receptors (TLRs), RIG-I-like receptors and DNA receptors (60). TLR-3, -7,- 8, and -9 are extremely important in sensing viral infections as they transduce signals to the system, which then activates antiviral processes. Agents that nonspecifically stimulate TLR-mediated responses may be potential antiviral remedies (60). In nonspecific immunity, antigen-presenting cells (APCs) and macrophages play important roles in cytokine secretion, antibody-dependent cell-mediated cytotoxicity and antigen presentation, processing and phagocytosis. The stimulation of naïve B cells and naïve T cells occurs through dendritic cells. Natural killer cells are upregulated, leading to the production of granulocyte-macrophage colony stimulating factor, tumor necrosis factor (TNF)-α, and INF-γ (61–63) (**Figure 1**).

## Natural Products as Immunomodulators

Normally, the body’s defense system maintains homeostasis; however, several endogenous or exogenous factors can alter its efficiency and effectiveness. Molecules from synthetic, natural, or biological origins can modify (suppress or stimulate) either the innate or adaptive component of immunity and are known as immunomodulators (63). Immunomodulators which improve the immune responses against infections either by activating or inducing the immune-system components are regarded as immunostimulants (64). In this review, the term immunomodulator refers to an immunostimulant. Some synthetic compounds and monoclonal antibodies are available as immunomodulators, but their use has been associated with a number of limitations, including side effects. For example, the use of recombinant cytokines as immunostimulants is associated with hypotension, myocardial infarction, cardiomyopathy and GI distress (63, 65). Thus, safe and effective agents are still required; indeed a significant amount of research has been conducted to identify natural products as immunomodulators. Immunomodulators from wide varieties of plants, their extracts, active plant ingredients and plant products have been investigated (66). Recently, plants containing polyphenolic compounds such as flavonoids in the form of fruit juice, extract, or isolated active constituents have been extensively studied for several beneficial effects, including their capability to modify the body’s defense system. Flavonoids are primarily antioxidant and exhibit inhibitory properties on inflammatory cytokine generation (67).

**Figure 2:**
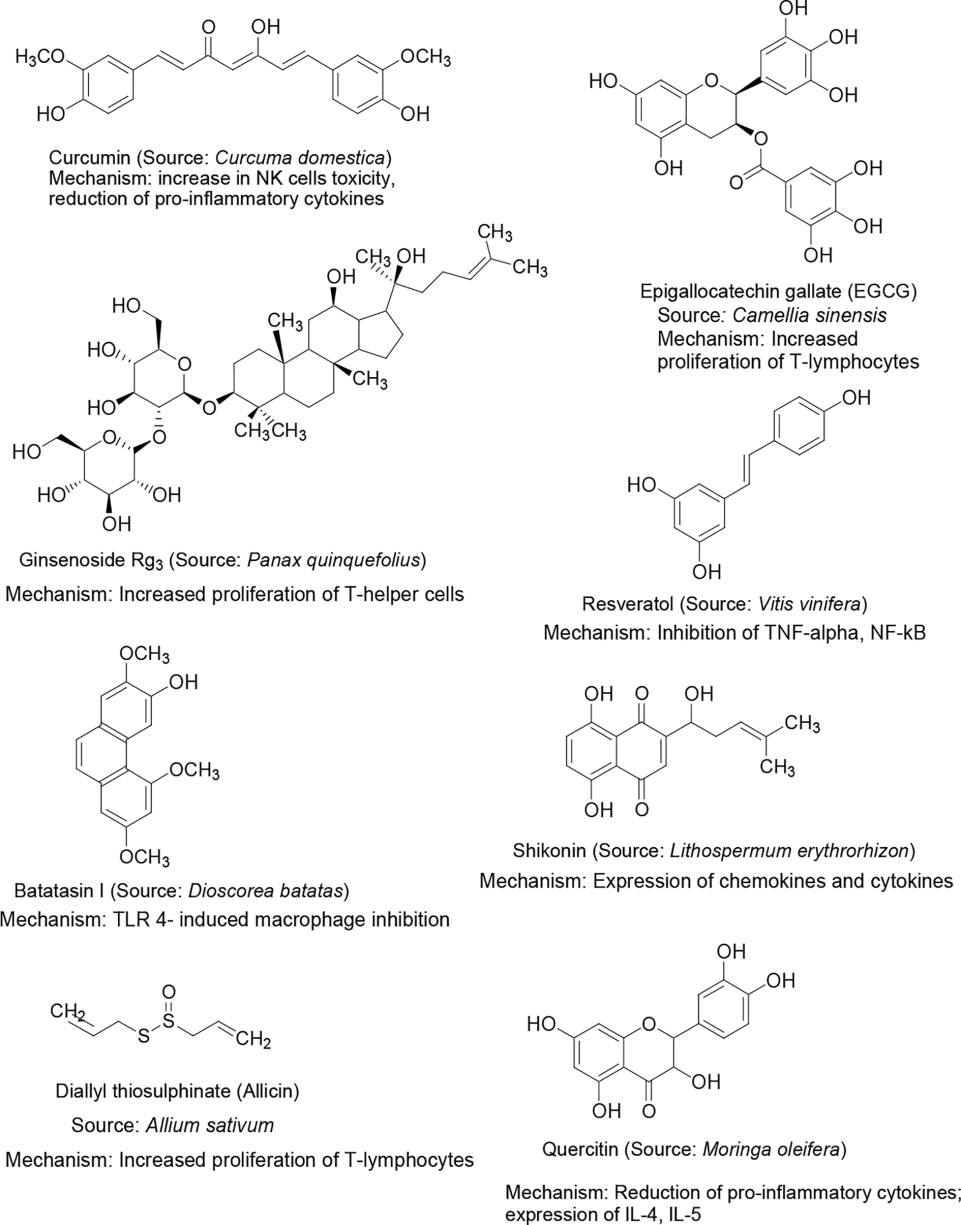
Chemical structure of selected natural bioactive compounds exhibiting immunomodulation activity, their major sources and mechanism of immunomodulation.

Several plant products have been investigated previously for direct treatment or as prophylaxis for infectious diseases (68, 69). Dietary supplements such as vitamins C, D and E are primarily antioxidants and are known to improve the immunity. The duration and severity of common cold has been shown to be reduced by a high dose of vitamin C and D supplements in patients (70, 71). Vitamin C has been included in at least 20 clinical studies to evaluate its efficacy in treating COVID-19 patients (72). Another ongoing trial is assessing the efficacy of vitamin A, B, C, D and E supplements on the improvement and mortality in COVID-19 patients (73). Vitamin E supplementation was found to improve cellular and humoral immunity and provides protection against infections (74); although, the degree of resistance is unclear. A clinical trial on elderly subjects revealed that long-term supplementation of vitamin E enhanced the resistance to mild respiratory viral infections, such as common cold (75). A large number of phytopharmaceuticals have been investigated for their beneficial effects in several diseases and many of them exerted significant immunomodulation properties. For example, resveratrol, an active constituent from grapevine, red wine, and peanuts, was found to show immunomodulation activity primarily through the inhibition of TNF-α or lipopolysaccharide (LPS)-mediated macrophages, NF-kB in phorbol 12-myristate acetate (PMA), dendritic cells, and myeloid (U-937) (63). Epigallocatechin-3-gallate (EGCG), a polyphenol constituent from green tea, reportedly exhibited immunomodulation activity primarily by blocking NF-kB activation. It also down-regulated nitric oxide (NO) production in macrophages and the expression of monocyte chemoattractant protein-1 (MCP-1), which is dependent on NF-kB inhibition (63). The squeezed sap of *Echinacea purpurea* showed nonspecific immunomodulation for use against mild to moderate respiratory infections, such as the common cold (70). Alkylamides are the principal active constituents, which act upon stimulation by the phagocytosis of macrophages and inhibition of inflammatory cytokine release such as TNF-α. It also increases circulating leukocytes, stimulates NK cells and possesses antiviral activity. Polysaccharide constituents were found to ameliorate the disease progress in mice infected with influenza (54) and were reported to improve the efficacy of influenza vaccine in immunosuppressed mice by activating the dendritic cells (76). Ginsenoside, the active component of ginseng, exhibited significant immunostimulant activity and was successfully used as an adjuvant with influenza vaccine (54, 77). Many of these phytochemicals have shown moderate to good immunomodulating activities in different ways in various *in vitro* and *in vivo* studies. Although, the results have been encouraging and favoring the use of these natural products as immunomodulators, high level systematic clinical studies are still warranted to establish their safety and efficacy. The structures of few important plant bioactive constituents exhibiting immunomodulation activity along with their sources are shown in **Figure 2**.

## Medicinal Plants Exhibiting Immunomodulation Activity

**Figure 3:**
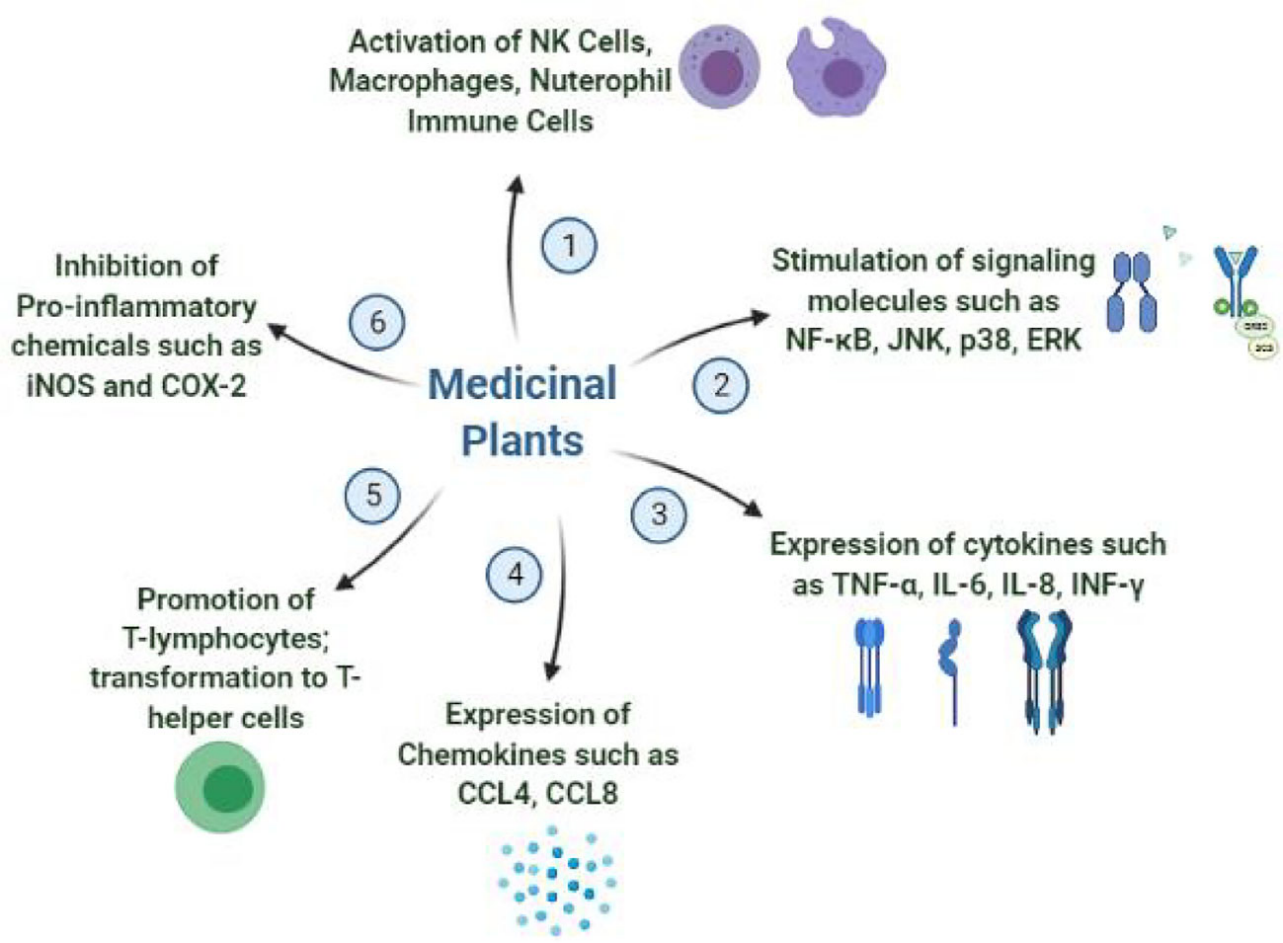
General immunomodulation mechanisms shown by medicinal plants.

The immunomodulating properties of medicinal plants have been studied much extensively during recent years due to the growing awareness on immune system modulation strategy to combat infectious diseases specifically the viral infections. A number of plants are already in use in folklore medicine for the treatment and prevention of viral infections either by directly affecting the pathogen or by stimulating the defense mechanism of human body in many ways. It is believed that the terpenoids present in these medicinal plants have promising efficacies in inhibiting the SARS-CoV-2 replications. Also, the alkaloidal structures such as lycorine, homoharringtonine and emetine have anti-coronaviral activities. Other isolated natural products such as emodin, baicalin, lguesterin, cryptotanshinone, silvestrol and sotetsuflavone showed inhibition of important viral replication enzymes inhibiting their growth (78). In this section, most important medicinal plants which have shown evidence based promising immunomodulation and antiviral activities in various *in vitro* and *in vivo* studies are summarized. In addition, other reported biological activities of the plant along with the mechanism by which they exert the immunomodulation activity are also discussed. **Figure 3** shows the mechanisms by which medicinal plants demonstrate the immunomodulation activity.

### *Echinacea purpurea* L.

*E. purpurea* L. is one of the most medicinally recognized plants and its products are commercially sold worldwide as a general health promoter and for its preventive actions on cold and flu. It shows effective antioxidant, anti-inflammatory, hypoglycemic, and antiproliferative activities. Root and above-ground parts extracts reportedly signal and stimulated immune responses by influencing macrophages, dendritic cells, monocytes, and NK cells and showed antiviral activity. The chemical constituents responsible for the antiviral activity and activating the body’s immune system include glycoproteins, soluble polysaccharides, phenolic compounds, caffeic acid derivatives, and alkylamides (79, 80). Other studies also showed that the preparations from plant extracts are among the most popular and high-demanding herbal product in USA as well as in other parts of the world due to its remarkable immunostimulatory activity and benefits in respiratory infections such as sore throat, cough, and cold (8, 81, 82). The oral administration of extract reportedly activates NK cells, polymorphic neutrophil immune cells, and macrophage phagocytic activity.

### *Dioscorea batatas* Decne

*D. batatas* Decne is extensively used in traditional and modern medicines and the tuber of the plant contains a number of active components, including dioscorin, diosgenin, mucopolysaccharides, batatasins, and glycoproteins, exhibiting immunomodulation activities upon oral administration. Tuber protein and dioscorin displayed mucosal and systemic immunomodulation activity through TLR4-induced macrophage activation by stimulating signaling molecules such as NF-kB, JNK, p38, and ERK, as well as by expressing cytokines such as TNF-α and IL-6 (8, 83). The ethanolic extract of bark inhibited iNOS and COX-2 expression in RAW 264.7 cells *via* NF-kB and ERK1/2, conferring anti-inflammatory activity (84, 85). Tuber extract also exerted immunomodulation effects by remarkably enhancing the granulocyte-macrophage colony-stimulating factor (GM-CSF) promoter activity in inflamed and normal skin (86, 87).

### *Artemisia annua* L

*A. annua* L. is a Chinese traditional medicine possessing artemisinin as the main active compound. Artemisinin is a sesquiterpene trioxane lactone primarily used as an antimalarial drug acting by disrupting the function of parasite mitochondria and by modulating host immunity. Artemisinin and its derivatives increase immune reconstitution and promote T cell activity; and are helpful in refurbishing immune function (88). In addition to antimalarial activity, it also showed beneficial effects in cancer treatment and inhibition of angiogenesis. Its tea infusion can be used for the treatment of malaria and HIV without any toxicity (89). Different phenolic compounds present in the extract showed activity against HIV and autoimmune diseases (8, 88) and was even found to be effective against the novel COVID-19 virus (90). Caffeic acid, quinic acid, artemisinin (antimalarial), scopoletin, melilotoside, casticin, artesunate, isovitexin, 3-caffeoyquinic acid, rosmarinic acid, and arteannuin B are some phytocompounds identified in tea infusion responsible for its activity (89, 91).

### Lithospermum erythrorhizon

*L. erythrorhizon* (Gromwell), a Chinese traditional medicine used from ancient times to treat smallpox, measles, macular eruptions, eczema, burns, and carbucles, is extremely popular in China and Taiwan. This herb is well known for the treatment of abnormal skin conditions such as wounds, inflammation, and burns. Dextrose capsule with 1.5 g of root extract improved stratum corneum hydration in parallel with increased ceramides in the outermost layer of epidermis (92). The primary active compounds isolated from root of the plant are Shikonin and derivatives, which are extensively studied primarily because of their remarkable antitumor and anti-inflammatory activities (93). The components also exhibited other pharmacological properties, such as antioxidant, anti-platelet, anti-atherosclerosis and immunomodulation. Shikonin is reported to express approximately 50 genes, including chemokines (CCL4 and CCL8), cytokines (IL-1b, IL-4 and TNF-α), and inflammatory modulators (PTGS2 and NFATC3) and reduced the surface expression of a primary HIV-1 co-receptor, CCR5, which may lead to the development of potential anti-HIV agents (94). Overall, the findings on the activities of Shikonin and derivatives at the cellular and molecular levels strongly indicated its capability to induce specific chemokines and chemotaxis in various immune-response cell types (8, 95, 96).

### Lycium barbarum

*L. barbarum*, characterized by bright orange red berries with yellow seeds, is rich in polysaccharides. Its glucosylated precursor, scopoletin, has carotenoids, flavonoids, and vitamins that are used as food supplements and therapeutic agents. Dietary polysaccharides can increase the ratio of probiotic bacteria, regulate the intestinal microenvironment such as decreasing the gut pH, and stimulating immunity such as macrophages or lymphocytes in intestinal mucosa. As a result, these polysaccharides are very helpful in fighting infections (97). Other studies reported that polysaccharides of the plant exhibit immunomodulation and antioxidant properties along with other various pharmacological activities (98). It activated local and systemic immune responses in H22 tumor-bearing mice, improved immune responses when used as an adjuvant with vaccines, and enhanced humoral immunity (99, 100)

### Ganoderma lucidum

*G. lucidum* is a white root fungus also known as Reishi mushroom or Red Lingzhi and has been used mainly in China for a long time to promote good health. The antiaging effect of its extract is primarily due to its antioxidant and immunomodulation properties. It is also reported to have anti-breast cancer and anti-neurodegeneration properties (101). Its polysaccharide, one of the most studied components, has been reported as an effective immunomodulator and potential inhibitor of tumor growth (102). The immunomodulation effects of this polysaccharide are broad spectrum and regulate the functions of the mononuclear phagocyte system, antigen-presenting cells, and cellular and humoral immunities (103). The antitumor activities of *G. lucidum* are also believed to be due to immunomodulation processes (104). Approximately 400 bioactive principles including polysaccharides, nucleotides, sterols, triterpenoids, steroids, proteins and, fatty acids have been identified. Formulations containing *G. lucidum* are approved by the Chinese FDA for the treatment of a large number of health-related problems including physical weakness, chronic hepatitis, chronic bronchitis, coronary heart disease, and anemia. The polysaccharide preparation is used for its antitumor, antioxidant, immunomodulation, and hypoglycemic activities (105).

### Eriobotrya japonica

*E. japonica* Lindl. is known as Loquat and used in Chinese folk medicine. The reported phytonutritional composition of its leaf and flower extracts is rich in phenolic and triterpenoid compounds, whereas the kernel is rich in proteins, minerals, starch, and tannins. It is cultivated commercially for its fruit, which is loaded with important nutrients such as carotenoids, flavonoids, vitamins, minerals, and antioxidants, known to have several health benefits (106). Loquat extract exhibited a wide range of medicinal properties, including those for the treatment of inflammation, diabetes, cancer, blood pressure regulation, immune-system improvement, viral infections, and has soothing effects on the respiratory system (107). The anti-inflammatory effects of Loquat are primarily due to its capability to reduce pro-inflammation mediators including iNOX, COX-2, IL-6, IL-8, TNF-α, and IL-1β and to enhance the generation of IL-10, an anti-inflammatory cytokine (106, 108).

### Agaricus blazei

*A. blazei* Murrill. is an edible mushroom possessing significant medicinal values owing to a number of chemical and nutritional constituents, such as polysaccharides, glycoproteins, phytosterols, β-D-glucan, and saponins. The polysaccharide components of the fungus are thought to be the main active component responsible for its antihyperlipidemic, immunomodulator, antioxidant, and anti-inflammatory activities (109). Its polysaccharide proved to be a promising adjuvant for cancer immunotherapy, when integrated with tumor-specific antigen into animal bearing ovalbumin-expressing tumors (110). Host immune activity was considerably boosted with specific stimulation of type 1 T-helper responses (110). With respect to immunomodulation effects, water and ethanol extracts from medicinal mushroom including *A. blazei* exhibited opposite effects on NK cells. Water extract increased the cytotoxic activity of NK cells by stimulating the expression of cytotoxic proteins NKG2D cell surface receptors and ethanol extract reduced the expression of cytolytic and cell surface receptors (111, 112).

### Dioscorea membranacea

*D. membranacea* Pirre is a Thai traditional medicine and one of the components for herbal formulation used to treat cancer and AIDS. Its rhizome extract enhances lymphocyte proliferation and NK cell activity, whereas the main constituent Dioscorealide B reduces these factors at high concentration (113). These findings indicated that the extract was an immunostimulant because of unknown compounds. It also exhibited antiallergy activity by suppressing β-hexosaminidase, TNF-α, and IL-4 in RBL-2H3 cells (114).

### Berberis vulgaris

*B. vulgaris* contains benzodioxoloquinolizine alkaloid (berberine), which is shown to significantly induce interleukin (IL)-12 production in a dose-dependent manner to counter the effects of HCV infection. IL-12 consequently increased the IFN-γ production and decreased the IL-4 level in antigen primed CD4^+^ T cells. Berberine also stimulates the T helper lymphocytes subset 1 (Th1) cytokine synthesis and decreases the Th2 subset (10, 115). The fruit of the plant have medicinal importance due to its antioxidant, anticancer, anti-inflammatory, antidiabetic, antibacterial, and hepatoprotective properties (116).

### Clerodendrum splendens

The polysaccharide obtained from the leaves of the plant *C. splendens* also exhibited potent immunomodulation activity. Its high-molecular-weight subfraction induced nitrous oxide and cytokine, TNF, granulocyte macrophage-colony stimulating factor, peripheral blood mononuclear cells, and monocytes with other wide-ranging agonist activities that considerably reduced disease severity (117, 118). Four clerodane diterpenes and phenyl propanoids from aerial parts showed antiproliferative activity (119).

### Tinospora crispa

*T. crispa* is a herbaceous vine with more than 65 different compounds including flavonoids, lactones, furanoditerpenes, alkaloids, lignans, and steroids, which have been isolated and used traditionally to treat a wide range of ailments (120). Magnoflorin, syringin, cardioside, quercetin, eicosenoic acid, and boldine are some of the active constituents of the plant with higher antioxidant potential than ascorbic acid and also responsible for activating the immune system by increasing the expression of IL-6, IL-8, and INF-γ. The plant extract was shown to increase the chemotaxis and phagocytic activity of macrophages and augmented the production of NO, ROS and pro-inflammatory cytokines (120). Studies suggested that *T. crispa* is a rich source of nutrients and antioxidants and showed hypoglycemic effects in an *in vivo* experimental study (121). The stem extract of the plant also showed application in prolonged recurrent malaria treatment at 100 and 200 mg/kg doses (122).

### Curcuma domestica

*C. domestica* (turmeric) is an Indian traditional medicine popular as dietary spice. Its polyphenolic compound, curcumin, is the main compound isolated from rhizome. Curcumin is available in various commercial preparations and effectively used in the treatment of several health problems. Curcumin has shown remarkable efficacy in the treatment of cerebral malaria through immunomodulation mechanisms (123). It is found to inhibit NF-κB activation and reduce pro-inflammatory cytokine production with expression of cytoadhesion molecules on endothelial cells (11, 124). Curcumin showed activity against various important human pathogens including influenza virus, HCV, HIV as well as SARS-CoV-2 (17). It can also block herpes simplex virus (HSV) type-2 (HSV-2) infection and abrogate the production of inflammatory cytokines and chemokines through genital epithelial cells *in vitro* (125, 126). The polyphenolic component also competently blocked Kaposi’s sarcoma-associated herpes virus replication and inhibited the pathogenic processes of angiogenesis and cell invasion (127). Different derivatives of curcumin were also reported to inhibit human influenza A viruses by blocking neuraminidase in a cellular and animal model (128).

### Achyranthes bidentata

Pretreatment with the immunomodulatory polysaccharide constituents of *A. bidentata* inhibited the proliferation of malaria parasite through immunostimulant mechanism. It was shown to strongly enhance the Th1 immune responses in body against the malarial parasites (129). Its combination with cubosome polysaccharides is found to be effective in promoting lymphocyte augmentation and in triggering the transformation of T-lymphocytes into T_h_-cells (130). Saponins from the plant have potential cellular and humoral immune responses with slight hemolytic and specific antibody enhancement (131, 132).

### Carica papaya

*C. papaya* Linn. seeds exhibited good antioxidant capacity and are proven to be beneficial in the treatment of type-2 diabetes mellitus by inhibiting α-amylase and α-glucosidase enzymes (133). The immunomodulation activity is shown by constituents pheophorbide A and saponin obtained from leaves, which exhibited cytotoxic activity on SCC25 cancer cells and stimulated cell- and antibody-mediated immunity, respectively (134, 135). Leaf juice of the plant has been shown to significantly increase the plasma CCL2/MCP-1 level to treat DEN-2 dengue virus (12). Aqueous fruit extract thereof improved the immune functions against acrylamide-induced oxidative stress (136).

### Morinda citrifolia

*M. citrifolia* L. reportedly showed immunomodulation and cytotoxic effects due to the presence of nordamnacanthal, which is an anthraquinone derived from fruits and stem of the plant. Moreover, an increase in the production of T helper, cytotoxic T1, and NK cells with a decrease in the size of tumors have been observed through *in vivo* studies (137). The plant exhibited a number of therapeutic effects including immunostimulant, anticancer, and anti-inflammatory. The fermented fruit extract was shown to restore skin-barrier-related proteins including filaggrin, loricrin, involucrin, zonula occludens-1, and occludin, and minimize 2,4-dinitrochlorobenzene (DNCB)-induced atopic dermatitis-like lesions through the modulation of skin barrier function and immune balance (138).

### Phyllanthus amarus

The methanol extract of roots of *Phyllanthus amarus* Schum. & Thonn. exhibited potent anticancer activity in MCF-7 cells through apoptosis induction mediated by improved intracellular reactive oxygen species (ROS) in conjugation with decreased mitochondrial membrane potential. Antioxidant, antidiabetic, antimicrobial, antiviral, anti-venom, and anti-inflammatory activities of the plant preparations have also been reported (139). Lignan-rich fractions from fruits showed potential to stimulate apoptotic cell death in cervical cancer cell lines *via* the commencement of P53 and P21 against DNA damage (140). Phytochemicals including corilagin, geraniin, gallic acid, phyllanthin, hypophyllanthin, ellagic acid, phyltetralin, niranthin, catechin, quercetin, astragalin, and chebulagic acid isolated from different plant parts are accountable for improving the innate and adaptive immune systems through different mechanisms (141).

### Azadirachta indica

*A. indica* (Neem) is extremely popular in many parts of the world with more than 140 biologically active components from different parts of the tree. This plant is traditionally used for its diverse medicinal properties, including anti-infective, anti-inflammatory, immunomodulatory, antioxidant, antiulcer, antimutagenic, and anticancer (142). The chemical constituents are mainly of two types: isoprenoids (azadiractins, salanin, vilasinin, and nimbin) and non-isoprenoids (polyphenolics, flavonoids, coumarins, and sulfurous compounds). The compound hyperoside isolated from leaves showed excellent interactions with conserved residues of nucleoprotein and could be a future drug for influenza virus (143). Approximately 35 components have shown significant influence as tumor suppressors by interfering with the carcinogenesis process in cervical, ovarian, and breast cancers (144). An experiment performed on mice has revealed that neem oil showed nonspecific immune stimulant activity *via* cell-mediated immune process. It also showed enhanced leukocyte cells, phagocytosis, expression of MHC class II antigens, gamma interferons, and lymphocytic proliferation (145).

### Chromolaena odorata

Polysaccharides isolated from the leaves of *C. odorata* (L.) showed significant antioxidant and immunostimulatory activities *via* the stimulation of peripheral blood mononuclear cells and production of IFN-γ in a dose-dependent manner. Apart from polysaccharides, the plant also contains alkaloids, sterol, polyterpenes, and flavonoids which exhibited a wide range of therapeutic profile, such as wound healing, cytotoxic, anti-inflammatory, and antioxidant (146). Flavonoids present in the plant may transform the appearance of glucagon-like peptide 1 and its release *via* Takeda-G-protein-receptor-5, thereby showing antidiabetic activity (147).

### Spatholobus suberectus

*S. suberectus* Dunn is a widely used traditional medicine for its therapeutic potential. Flavonoids, the major bioactive compounds of the plant reportedly reduced the PCV2-infected oxidative stress and exhibited immunosuppression. They were found to enhance the spleen and thymus indices such as SOD activity, GSH level, and GSH to GSSG ratio, as well as the downregulation of XOD and MPO activities. This finding indicated that the plant might act as a good antioxidant for treating diseases associated with oxidative stress including viral infections (148, 149). The dried vine stem of the plant also showed good anti-inflammatory activity by reducing the mRNA expression of some pro-inflammatory cytokines such as TNF-α, iNOS, and COX-2 (150).

### Quillaja saponaria

*Q. saponaria* contains saponins as its major component, which modulated immune responses in clinical and preclinical studies and is useful as a vaccine adjuvant. Its action is attributed to the activation of cytotoxic T lymphocyte (Th1) and cytokine (Th2) production. The bark of the plant also exhibited antitumor, hepatoprotective, antiviral, antibacterial, and antiparasitic potential (151). As hepatoprotective agent, bark saponin decreased the iron-induced elevation of ALT, AST, ALP, GGT, LDH, MDA, NOx, TC, and TG, as well as the total direct and indirect bilirubin and albumin levels (152).

### Arisaema jacquemontii

*A. jacquemontii* Blume is mainly found in the Himalayan region and the hexane extract of the plant exhibited remarkable antioxidant and immunomodulation activity comprising both stimulation and suppression. Immune stimulation was based on humoral and DTH responses, and immunosuppression was due to the mitogen-induced proliferation of T and B cells (153). The phenolic and flavonoid compounds present in ethanol and methanol extract were responsible for a wide range of pharmacological activities, including antioxidant, antileishmanial, anti-infective, and protein-kinase inhibition, whereas *n*-hexane and chloroform extracts exhibited high cytotoxicity against prostate cancer (154–156).

### Radix Astragali

*R. Astragali* reduced the immunosuppression induced by methotrexate in mouse spleen cells. The cell proliferation was suppressed by the plant extract, and the immunomodulation effect was demonstrated through the enhancement of IL-1α and IL-12p40 mRNA expression (157, 158). Plant products in combination with *Radix Angelicae Sinensis* exerted a synergistic effect on the immunological balance of T lymphocytes (159).

### Nymphaea rubra

Purified polysaccharides of *N. rubra* Roxb showed immunomodulation activity with stimulating effect on immature dendritic cells and promotion of the secretion of T_R_1 cytokines (160, 161). Its young leaves, peduncle, and rhizomes were found to be rich in proteins (20.5%–43.5%) and carbohydrates (63.14%), making it valuable remedy to treat diarrhea, piles, and cough (162).

### Dimocarpus longan

Longan polysaccharide 1 from fruit pulp of *D. longan* significantly stimulated the production of cytokine interferon-γ, enhanced the activity of murine macrophages, and improved B-and T-lymphocyte production to inhibit tumor formation combined with immunomodulation effect (163, 164). Moreover, the polysaccharide–protein complex from fruit pulp showed strong immunomodulation activity (165).

### Camellia sinensis

*C. sinensis*, popularly known as green tea, has been extensively used worldwide as a traditional medicine since ancient times owing to its antioxidant property. Catechins including epigallocatechin gallate (EGCG), epigallocatechin (EGC) epicatechin (EC), and EC gallate (ECG) are the major bioactive molecules isolated from tea leaves and are responsible for its antioxidant, antiviral, anticancer, and antifungal activities. EGC and EGCG are reported to exhibit immunomodulation activity through the generation of cytokines and T lymphocyte proliferation. The extract also reportedly enhanced the production of lymphocyte, monocytes, IL-1α, and IL-1β (63, 166). Non-catechin flavonoids from seeds improved TNF-α-impaired insulin, stimulating glucose uptake and insulin signaling with anti-metabolic syndrome and anti-inflammatory properties (167). Sapogenins, glycosides, and organic acids are triterpenoid saponins from tea leaves that reportedly inhibit human ovarian cancer cells selectively by mediating apoptosis through the extrinsic pathway and initiating anti-angiogenesis (168). Conversely, aflavin polyphenol compounds obtained from black tea are anti-HSV-1 agents (169).

### Aronia melanocarpa

*A. melanocarpa* contains polyphenolic compounds, procyanidins and anthocyanins in its berries and bark which exert immunomodulation and anti-inflammation activity by inhibiting nitric oxide production (170). Reduction in blood glucose level and antioxidant properties are observed through the inhibition of alpha-glucosidase and xanthine oxidase, respectively (171, 172). Along with polyphenols, flavonoids such as anthocyanins, proanthocyanidins, flavanols, flavonols, cynidin-3-galactoside, and cyaniding-3-arabinoside exhibited wide range of therapeutic benefits, including gastroprotective, hepatoprotective, antiproliferative, and cardiovascular-protective properties (173).

### Chenopodium quinoa

Polysaccharides obtained from the seeds of *C. quinoa* Willd. are considered to be excellent dietary sources of natural antioxidants promoting human health through immune-system modification (174). Purified polysaccharides from the plant have been further reported to exhibit immunomodulation activity by increasing macrophage proliferation and suppression of NO production on inflammatory RAW264.7, in addition to antioxidant and anticancer properties (175). Quinoa saponins such as phytolaccagenic, oleanolic and serjanic acids, hederagenin, 3β,23,30 trihydroxy olean-12-en-28-oic acid, 3β-hydroxy-27-oxo-olean-12en-28-oic acid, and 3β,23,30 trihydroxy olean-12-en-28-oic acid exhibit molluscicidal, antifungal, anti-inflammatory, hemolytic, and cytotoxic activities (176).

### Abrus cantoniensis

*A. cantoniensis* is a Chinese folk medicine and vegetable (Hance); the isolated polysaccharide fractions of which showed significant immunomodulation and antitumor properties. The fraction activated lymphocyte proliferation, stimulated the production NO of peritoneal macrophages, and exhibited inhibitory effects on the migration of MCF-7 cells (177). The whole plant showed considerable wound-healing capacity at a concentration of 5% (v/w) as evidenced by increased wound contraction, hydroxyproline content, and decreased epithelialization time compared with control (178).

### Rhizoma gastrodiae

The rhizomes of *R. gastrodiae* have been used to treat convulsions, hemiplegia, neurasthenia, headache, ischemia, and dementia. Polysaccharides, namely, gastrodin, 4-hydroxybenzyl alcohol and parishin B are the main phenolic compounds present in its rhizomes. They were shown to stimulate the macrophages to release NO and enhance phagocytosis in a dose-dependent manner, producing immunological activity (179). The antidepressant effect of the drug is probably exhibited by the modulation and regulation of its monoamine oxidase (MAO) activity and monoamine concentration. Furthermore, its antioxidant activity, GABAergic system induction, neuroprotection and anti-inflammatory properties have also been reported (180, 181). Acidic polysaccharides reduced hypertension and improved serum lipid levels in rats fed with high-fat diet (182).

### Hypoxis Hemerocallidea

African potato (*H. Hemerocallidea*) is one of the most popular medicinal plants in South African region as a remedy for several diseases. The plant is primarily used for immunomodulation activity to treat diseases occurring when the body immunity is weakened, such as in HIV/AIDS, cancer, flu, common cold, and rheumatoid arthritis. Gold nanoparticles prepared from aqueous extracts of the plant and its glycoside content hypoxoside showed immunomodulation effects *via* reduction in cytokine levels in macrophages and NK cells, indicating their potential as an anti-inflammatory agent (183). Hypoxoside isolated from the plants is frequently used as an immunity enhancer in South Africa. Rooperol, a hydrolytic product of hypoxoside, demonstrated antioxidant activity by inducing the production of NO and ROS and phagocytosis (184).

### Panax quinquefolius

*P*. *quinquefolius* (American ginseng) plant extract exerted powerful immunomodulation effects on innate and adaptive immunity in healthy mice, apart from showing potential antioxidant activity to combat the oxidative stress (185, 186). The plant extract also showed reduced incidence, duration, and severity of flu and colds in controlled clinical trials involving healthy and infected individuals (187, 188). Among herbal supplements, ginseng is one of the most extensively studied for its beneficial effects and few toxic effects. Clinical substantiation showed its efficiency in specific disease treatments, such as dementia, diabetes mellitus, respiratory infections, and cancer (187, 189).

### Macrocystis pyrifera

Fucoidans are polysaccharides in *M. pyrifera* containing sizable contents of *L*-fucose and sulfate ester moieties with wide-ranging biological activities, such as antiviral, antitumor, immunomodulation, and antioxidant. Fucoidans from *M. pyrifera* have potent immune-activating effects by influencing human neutrophils, NK cells, dendritic cells, and T cells to enhance antiviral and antitumor responses (190).

### Orthosiphon stamineus

*O. stamineus* is an important medicinal plant containing a number of phytochemicals including terpenoids, flavonoids, and essential oils, which are responsible for a wide range of traditional and therapeutic uses of the plant (191). The leaf extract of the plant exhibited promising anti-infective potential by influencing the immune mechanism of the host cells by significantly restoring the suppressed lys-7 defense gene expression in nematodes infected with *Staphylococcus aureus* (192). In another study, *O. stamineus* exerted considerable antioxidant and immunomodulation effects and antibacterial potential against gram-positive bacteria. The leaf extract also exhibited stimulatory action on human peripheral blood mononuclear cells and could thus be used to increase immunity and avoid ROS related disorders (193).

### Hedysarum polybotrys

*H. polybotrys* extract contain formononetin and proanthocyanidin as chemical constituents which showed remarkable immune stimulation activity. The proliferation of splenocytes and LPS-activated RAW 264.7 cells were stimulated and NO radical scavengering was increased, whereas NO-induced cytotoxicity was decreased. Conversely, the extract also inhibited PGE2, COX-2, iNOS, NO, LPS-activated 264.7 cells, and splenocytes (194). This finding suggested that the extract can be used when immunity stimulation and anti-inflammatory therapy is required (195). Furthermore, a wide range of chemical constituents are present in the plant, making it therapeutically important to treat several diseases, including viral infections and cancers (196).

### Momordica charantia

*M. charantia* is a subtropical vegetable with remarkable medicinal value and is utilized as a traditional remedy for the treatment of various diseases. Polysaccharides from its fruits showed a significant increase in serum hemoysin production, NK cell cytotoxicity, carbolic particle clearance index, thymus index, and spleen index in cyclophosphamide-induced immunosuppression in mice (197). The proliferation of normal and concanavalin A-induced splenic lymphocytes was also stimulated, showing its potential as an immunotherapeutic adjuvant (197). The methanol and diethyl ether extracts of M. *charantia* leaves exhibited significant therapeutic potential against *S. typhi* infections owing to immunomodulation activity (198). The extract also increased the antibody production and leukocyte mobilization against the infection.

### Ficus aurantiaca

Triterpenoids isolated from stem of *F. aurantiaca* Griff have been investigated for inhibitory action on polymorphonuclear leucocyte chemotaxis and the production of neutrophil and whole-blood ROS. These components might act as potential lead molecules for the development of new immunomodulation agents for the innate immune response of phagocytes (199).

### Gynostemma pentaphyllum

*In vivo* studies on *G. pentaphyllum* showed that its polysaccharides effectively increased the spleen and thymus indices, activated macrophage phagocytosis and NK cells, and exhibited activity on Con A/LPS-stimulated splenocytes in a dose-dependent manner. It also increased the IL-2 levels in sera and spleen, as well as the SOD, GSH-Px, T-AOC, GSH, and CAT levels; conversely, MDA levels were decreased (200). Aerial parts showed promising prospects as purposeful food and nutraceuticals owing to their numerous biological activities including antioxidant, immunomodulation, and antitumor (201). Multiple mechanisms of action have been proposed regarding the anticancer activities of plants including immunomodulating action (202).

### Astragalus membranaceus

*A. membranaceus* is used as traditional medicine to treat cold and flu and is also administered as health tonic. The plant reportedly boosted the immune system, which was attributed to its major constituents, polysaccharides, flavonoids, amino acids, and minerals (188, 203). Another study reported that the oral administration of polysaccharides from A. *membranaceus* dose-dependently inhibited tumor growth and protected the immune system by promoting macrophage pinocytosis. Thus, the lymphocyte subsets in the peripheral blood of tumor-bearing mice increased (204). Several other studies showed it as an anti-inflammatory and antioxidant remedy against inflammation and gastrointestinal diseases (205, 206). The plant was also found to reduce intestinal mucosal damage and promoted tissue repair by inhibiting the expression of inflammatory cytokines (207).

### Zizania latifolia

*Z. latifolia* is a traditional medicine also known as Chinese wild rice. It is rich in proteins, minerals, vitamins, and bioactive substances, such flavonoids, polysaccharides, and saponins, exhibiting therapeutic potential including immunomodulation and antioxidant activities and shows effectiveness in treating various diseases. Water-extractable polysaccharides isolated from swollen culms reportedly exhibited potent immunostimulation by increasing the proliferation, phagocytosis, and NO production of murine macrophages RAW 264.7. The plant has the potential to be considered as a promising immunomodulator in the field of medicine and functional foods (208, 209).

### Moringa oleifera

*M. oleifera* is extensively utilized as food and medicine because of its wide range of nutritional and therapeutic potential including immunostimulatory effects. These effects are attributed to a number of bioactive principles, such as vitamins, flavonoids, minerals, isothiocyonates, and polyphenols present in the leaf, pod, seed, and bark of the plant (210). Several studies reported that the leaf extract stimulated humoral and cell-mediated immunity and might be useful as an alternative remedy under immunosuppressed conditions (211). The extract showed an increase in lymphocyte, neutrophil, and WBC count in cyclophosphamide-immunosuppressed rats, in addition to enhanced hemagglutination antibody titer in sheep red blood cells (212, 213). The plant is widely consumed in Uganda as an immunity booster to treat several diseases (210, 213). The leaf extract inhibits NF-kB action and NF-kB-dependent downstream events, thereby reducing the inflammatory processes and restoring the antioxidant status in mice fed with high-fat diet (214).

### Allium sativum

*A. sativum L.* (Garlic) is frequently consumed as a spice worldwide and as a traditional medicine in China and India owing to its diverse health benefits. The therapeutic effects of the plant is attributed to the presence of a number of bioactive constituents, including organosulfurs, saponins, and polysaccharides. The strong immunomodulation activity of garlic is primarily because of its polysaccharide contents, which regulate interferon-γ, TNF-α, IL-6, and IL-10 in macrophages (215). Garlic extracts regulated immune-system homeostasis and maintained immune responses mainly by the expression and proliferation of cytokine genes for instance; diallyl trisulfide content present in garlic increased the T lymphocyte proliferation in mice. The protein fraction of garlic was reported to enhance the cytotoxicity of macrophages and lymphocytes (216). The occurrence and severity of flu and cold is also considerably reduced by the intake of aged garlic extract through immune-system improvement (217).

### Emblica officinalis

*E. officinalis* (Amla) was shown to restore the Cr-induced immunosuppression by re-establishing the proliferation of lymphocytes and the production of IL-2 and INF-α (141). Furthermore, the fruit extract restored the antioxidant status to normal by inhibiting the Cr-induced production of free radicals and reversing Cr-induced DNA fragmentation and apoptosis in an *in vitro* experiment (218). Amla is extremely rich in vitamin C (even more than orange or lemon) and has been traditionally used as a tonic to recover vigor and lost energy. It also contains other bioactive constituents such as alkaloids, phenolic compounds, and tannins and exhibit a wide range of therapeutic actions, including antidiabetic, antioxidant, and anticancer. Amla stimulated the NK cell activity and antibody-dependent cellular cytotoxicity, enhancing 35% life span of tumor-bearing mice (219, 220). The immunomodulation mechanism of amla in reducing arsenic-induced oxidative damage and apoptosis in mice was also reported (221).

### Origanum vulgare

Peppermint oregano (*O. vulgare*) is a popular herb in the mint family known for its important medicinal qualities. Carvacrol, the main constituent of essential oil, offered good antiviral properties against murine norovirus in an *in vitro* study (222). It also exhibited antiviral properties against HSV type-1 (HSV-1), rotavirus, and respiratory syncytial virus (RSV) (223).

### Salvia officinalis

Stems and leaves of sage (*Salvia officinalis*) contain safficinolide, α-pinene, and β-myrcene which were derived from its volatile oil and showed good antiviral activities against HIV type-1 (HIV-1), HSV-1, and Indiana vesiculovirus (224). Another *ex vivo* study on the leaf extract of the plant showed presence of triterpenic compounds and verbascoside which were responsible for its immunomodulation activity (225).

### Osimum basilicum

Basil (*O. basilicum*) (Tulsi) is rich in compounds such as apigenin and ursolic acid, exhibiting potent antiviral activity against herpes, hepatitis B, and enterovirus. Aerial parts of Tulsi increased the levels of helper T cells and NK cells to boost the immune system (226, 227). Trans anethole from the plant has powerful antiviral effects against herpes viruses, as well as potent immunomodulation activity (228).

### Other Plants

There are a good number of other medicinal plants which have shown good to moderate immunomodulation and antiviral activities in various *in vitro* and *in vivo* models. For instance, oleanolic acid in rosemary (*Salvia rosmarinus*) has been proven to have good effect against herpes, HIV, influnza, and hepatitis A viruses (229). Sambucus, licorice, ginger, ginseng, and dandelion showed dominant antiviral activities in many laboratory experiments. Glycyrrhizin and lycorine from *G. glabra* and *Lycoris radiate*, respectively, showed good activity against SARS-CoV (230). Glycyrrhizin has even showed good potential against the COVID-19 virus SARS-CoV-2 and may emerge as an alternative drug for its treatment (231). Based on these findings, a novel combination of vitamin C, curcumin and glycyrrhizic acid (VCG plus) was proposed for the treatment of coronavirus infections (232). This combination is believed to regulate the innate immune response. Many other plants such as *Centella asiatica*, *Zingeber officinale*, *Andrographis paniculate*, *Panax ginseng*, *Trigonella foemnum graecum*, *Picrorhiza scrophulariiflora*, *Phyllanthus debilis*, *Baliospermum montanum*, *Tinospora cordifolia*, *Curcuma longa*, and *Pouteria cambodiana* reportedly displayed either immunostimulant or immunosuppressive properties by modulating acquired or innate immune responses (199). A number of single and compound drugs are advised in the Unani medicine based on the medicinal plants which are claimed to be antiviral, antipyretic, blood purifier, cardioprotective and expectorant activities and can be effective in the treatment and management of viral infections including COVID-19 (233).

The immunomodulation potential exhibited by plant extracts or active compounds offered novel sources of lead compounds for the development of new molecules intended to be used for treating various diseases including viral infections through immunotherapy. Apart from the plants mentioned in this review, numerous other plants also exhibited immunomodulation properties which might prove effective in fighting viral infections as direct or prophylactic therapy. The important plants showing immunomodulation activity, along with their regional sources, bioactive constituents, and other reported biological activities, are listed in **Table 1**.

**Table 1:** List of selected plants demonstrating immunomodulation activity, source countries, parts used, their bioactive chemical constituents and other reported activities.

Plant name (family)	Native country	Part used	Bioactive constituents	Other reported activities	References
**(A) Plants having polysachharides as major bioactive constituent**					
*Echinacea purpurea* L. (Asteraceae)	United States and Europe	Aerial part and roots	Polysaccharides, Alkamides, caffeic acid derivatives and lipoproteins	Wounds, toothaches, respiratory infections, colds, sore throat, cough and burns	8
*Lycium barbarum* (Solanaceae)	China, Asia, Europe	Fruits, roots, leaves	Polysaccharides, glucosylated precursor, scopoletin, carotenoids, flavanoids, amino acids, vitamins and minerals	Antioxidant, antiaging, antitumor, anti-inflammatory, hypoglycemic, hypolipidemic, cardioprotective	99
*Ganoderma lucidum* (Ganodermataceae)	China, Japan, Korea	Fruits	Polysaccharides (β-*D*-glucans), ganodermasides, triterpenoids, sterols, steroids, proteins and fatty acids	Antitumor, anti- ageing, antioxidant, neuroprotection, treatment of chronic hepatitis, ulcers, chronic bronchitis etc.	105
*Agaricus blazei* Murrill (Agaricaceae)	Brazil	Fruits, mycelium	Polysaccharides, sterol, ergosterol, potassium	Antihyperlipidemic, antioxidant, anticancer and anti-inflammatory activities.	109
*Clerodendrum splendens* (Lamiaceae)	Western Africa	Leaves, bark, flowers	Polysaccharides, arabinogalactan, terpenoids, flavanoids, volatile oils, cyanogenic glycosides, alkaloids, tannins, saponins, phenols	Treatment of malaria, coughs, venereal infections including gonorrhea and syphilis, skin diseases, ulcers, asthma, and uterine fibroid	118
*Achyranthes bidentata* (Amaranthaceae)	India, China Japan, Nepal	Aerial parts, roots	Polysaccharides, saponins	Anti-malarial, diuretic, antiviral, toothache	129, 132;
*Morinda citrifolia* L (Rubiaceae)	Southeast Asia, Australasia	Fruit, stem, leaves, roots rhizomes	Carbohydrates dietary fibers, minerals, vitamins, lignin, catechin, polysaccharides, flavanoids, alkaloids,	Skincare, anti-asthma, anti-diabetic, anticancer, immunomodulator, anti-inflammatory, antihypertensive, anti-infective	138
*Nymphaea rubra Roxb* (Nymphaeaceae)	India, Australia, Africa America	Rhizome, leaves, flower, peduncle	Polysaccharides, proteins	Cough, piles, diarrhea	161, 162
*Dimocarpus longan* (Sapindaceae)	Asia, China, Thailand	Fruit	Polysaccharides, proteins	Improves immunity, antioxidant	164, 165
*Abrus cantoniensis* (Fabaceae)	China	Aerial parts	Polysaccharides, Polyphenols	Anti-tumor activity, wound healing	177, 178
*Rhizoma gastrodiae* (Orchidaceae)	China, Taiwan, Japan, Korea	Rhizome	Polysaccharides, Polyphenols	Headache, dizziness, liverprotective, paralysis, rheumatism, limbago pain, migraine, anti-spasmodic, joint and nerve disorders, antidepressant	179, 182
*Momordica charantia* (Cucurbitaceae)	Asia, Africa, China	Fruit, seeds, roots, leaves,	Polysaccharides, charantins, alkaloids, triterpenoids, phenolic compounds	Anti-diabetes, hypolipidemic, antitumor, anti-inflammatory, anti-obesity, antiviral, antioxidant	197
*Gynostemma pentaphyllum* (Cucurbitaceae)	Asia, China, New guinea, Japan, Korea	Stem, rhizome, fruits	Polysaccharides, Sterols, saponins, flavonoids, gypenoids,	Antioxidant, immunomodulatory, antitumor, hepatoprotective and neuroprotective.	200, 201
*Astragalus membranaceus* (Fabaceae)	China,	Leaves, aerial parts	Polysaccharides, flavonoids, minerals, amino acids, triterpenoids, glycosides	Antitumor, anti-inflammatory, antiviral, Treat cold and flu allergies	188, 206
*Zizania latifolia* (Gramineae)	China, Japan, Korea	Rhizome, stem, leaves, seeds	Polysaccharides, proteins, minerals, and vitamins, flavonoids, saponin, and phytosterol, anthocyanins	Antioxidant, diabetes, obesity, and cardiovascular diseases	208
*Macrocystis pyrifera* (Laminariaceae)	Pacific ocean, Southern ocean, North coast of America	Whole plant	Polysaccharides, Iodine, potassium,	Antiviral, antitumor, immunomodulatory, antioxidant	190
**(B) Plants having alkaloids as major bioactive constituent**					
*Dioscorea batatas* (Dioscoreaceae)	East Asia, China	Tubers	Dioscorin, diosgenin, mucopolysaccharides, batatasins and glycoproteins	Treatment of asthma, chronic diarrhea, poor appetite, diabetes, emotional instability, inflammation and uncontrollable urination	8
*Berberis vulgaris* L. (Berberidaceae)	Europe, Africa, USA, New Zealand	Root and bark	Berberine, berbamine, palmatine	Anti-cancer, antioxidant, anti-inflammatory, anti- diabetic, GIT disorders, cough, acne, tonic	116
*Phyllanthus* amarus Schum. & Thonn. (Euphorbiaceace)	America, Africa, Australia, Asia	Leaves, aerial parts, roots	Alkaloids, flavonoids, tannins, lignans, sterols, triterpenes, volatile oils	Antioxidant, Cold, flu, anti-infective, antiviral, anticancer, antidiabetic, jaundice, antimalaria, GIT disorders, inflammation	139
*Camellia sinensis* (Theaceae)	China, India, Nepal	Leaves, aerial parts	Caffeine, vitamins, tannins, minerals, flavonoids, epicatechins	Antioxidant, diuretic, migraine, headache, anti-obesity, anticancer, diabetes, CNS stimulant, anti-inflammatory, antiviral	166, 167, 169
*Moringa oleifera* (Moringaceae)	America, Taiwan, India	Root, seeds, leaves, oil, fruits, seedpods, flowers	Alkaloids, vitamins, isothiocyanates, minerals, dietary fiber, poly phenols, saponins	Antioxidant, antidiabetic, anti-inflammatory, anticancer, hypolipidemic, hepatoprotective, anti-hypertensive	210
*Hypoxis Hemerocallidea* (Hypoxidaceae)	Africa, Mozambique, Zimbabwe	Aerial parts, whole plant, corms	Alkaloids, glycosides, carbohydrates,	Antiinflammatory, anti-hypertensive, anti-diabetic, antiulcer, antibacterial, antioxidant, anticancer, antiviral.	183
**(C) Plants having phenols and polyphenols as major bioactive constituent**					
*Eriobotrya japonica* Lindl. (Rosaceae)	China, Japan, Turkey, Brazil, India, Pakistan, Italy, Spain, Israel	Leaves, fruits	Phenolics, terpenoids, flavonoids, carotenoids, organic acids, vitamins, sugars	Antioxidant, anticancer, anti-inflammatory, anti-diabetic, hypolipidemic, anti-aging, antiallergic and anti-infective	106
*Curcuma domestica* (Zingiberaceae)	Asia, China	Rhizome	Curcumin	Antioxidant, anti-malarial, anti-diabetic, anticancer, anti-infective activities	11
*Chromolaena odorata* (L.) (Compositae)	Europe France, Thailand, china	Leaves, seed oil,	Polyphenols, tannins, saponins, sterols, polyterpenes, alkaloids, flavonoids	Wound healing, anti-inflammatory, antidiarrheal, antioxidant, analgesic, cytotoxic, antibacterial	146
*Arisaema jacquemontii* Blume (Araceae)	Afghanistan, East Asia, France	Tuber, leaves, roots	Phenols, flavonoids, alkaloids, saponins, tannins, sterols, protein, carbohydrates, fat	Cytotoxic, anti-fungal, antimicrobial, antioxidant, antieishmanial, protein kinase inhibition, anti-prostate cancer activities	156
*Aronia melanocarpa* (Rosaceae)	America, Georgia, Alabama, Arkansas	Fruits, leaves	Polyphenols, anthocyanins, flavonoids	Immunity, antioxidant, diabetes, inflammation,	171–173
*Zingiber officinale* (Zingiberaceae)	Asia, Hawaii, Europe, Roman, Middle east	Rhizome	Volatile oil, Zingerone, shogaols, gingerols, gingerdione, acetoxy-6-dihydroparadol	Antimicrobial, antidiabetic, anti-inflammatory, antioxidant, hepatoprotective, nephroprotective	234
**(D) Plants having sesquipterpene as major bioactive constituent**					
*Artemisia annua* L. (Asteraceae)	China, Asia, North America	Aerial parts	Artemisinin, dihydroartemisinin, artemether, arteether, artesunate, artelinate, caffeic acid, quinic acid, casticin, artesunate, isovitexin, 3-caffeoyquinic acid, rosmarinic acid, arteannuin B	Malaria, inflammation, antioxidant, antibacterial, hepatoprotectant, antihistaminic, anti-HIV bronchitis, cancer, hemorrhoids, autoimmune disorders	91
*Dendrobium nobile* (Orchidaceae)	China, India, Bangladesh, Assam, Nepal, Bhutan, Hawaii	Stem, flowers, leaves	Sesqueterpenes, Longifolene, 1-Heptatriacotanol, Z,Z-6,28- Heptatriacotantadien-2-one, Dendroban-12-one,	Antiviral, anticancer, antimicrobial, antioxidant, analgesic and antipyretic	235
**(E) Plants having terpenes and tritepenoids as major bioactive constituent**					
*Carica papaya* Linn. (Caricaceae)	India, Sri Lanka, Australia, Malaysia, Vietnam	Seed, leaves, fruit	Saponins, flavanoids, phenols, terpenoids, steroids, alkaloids	Antidiabetic, anticancer, antipyretic, antimalarial, dengue fever, beriberi, asthma	133, 134
*Azadirachta indica* (Meliaceae)	India, Africa, Australia, middle east,	Leaves, bark, fruit, wood, roots, flowers, oil	Nimbidin, nimbini azadirone, azadiractins, salanin, vilasinin, nimbin etc, polyphenolics, flavonoids, coumarins, sulfurous compounds	Anti-infective, anti-inflammatory, antioxidant, anti-ulcer, anti-mutagenic, anticancer, antiviral etc.	143
*Quillaja saponaria* (Quillajaceae)	Europe, Brazil, UK, USA, Japan, China	Bark, root, wood	Saponins, quilaic acid, gypsogenin, phytolaccinic acid,	Antidandruff, anti- bacterial, antiviral, anti-parasitic, antitumor, hepatoprotective, immunoadjuvant activities	151
*Chenopodium quinoa Willd.* (Amaranthaceae)	Northwestern South America, USA, Kenya, India, Europe,Aurtralasia	Seeds, leaves	Saponins, polysaccharides, polyphenols, phytosterols, proteins, vitamins, minerals, fat, flavonoids,	Antiseptic, antioxidant, antidiabetic, anti-apoptotic, antiobesity, neuroprotective, hemolytic, antiviral	174, 176
*Panax quinquefolius* (Araliaceae)	USA, Canada, China	Roots, rhizome, leaves	Ginsenosides, protopanaxadiol, protopanaxatriol	Treat dementia, diabetes mellitus, respiratory infections and cancer.	187
*Orthosiphon stamineus* (Lamiaceae)	Tropical areas, South Asia	Leaves, aerial parts	Terpenoids, flavonoids, caffeic acid derivatives, essential oil,	Antimicrobial, antioxidant, hepatoprotection, antigenotoxic, antiplasmodial, cytotoxic, cardioactive, antidiabetic, anti-inflammatory activities.	191
*Ficus aurantiaca Griff* (Moraceae)	Singapore	Stem, leaves, fruits,	Triterpenoids, Coumarin,	Headache, wound and toothache	199
*Actaea racemosa* (Ranunculaceae)	America, Georgia, Missouri, Aransas	Roots, rhizome	Triterpene glycosides, 27-deoxyactein	Dietary supplement, treatment and management of menstrual and menopausal symptoms	236
*Gymnema sylvestre* (Apocynaceae)	Asia, China, Arabian peninsula, Africa, Austratia	Leaves,	Gymnemic acids, triterpenoids saponins, flavonoids, tannins, gymnemanol, gurmarin	Antidiabetic, anti-infective, anti-asthma, anticancer, anti-inflammatory, anti-hypercholesterolemia, antiviral, cardiovascular diseases	237
*Rapanea melanophloeos* (Primulaceae)	South Africa,	Fruits and seeds	Terpenoids, alkaloids, saponins, glycoside, tannins, flavonoids	To treat fever, chest disease, cough, antiviral	238
**(F) Plants having flavanoids and isoflavanoids as major bioactive constituent**					
*Tinospora crispa* (Menispermaceae)	South east Asia, Africa, Thailand, Malaysia, Indonesia	Stem, seed,	Flavanoids, terpenoids, alkaloids, glycosides lignans, steroids	Anti-inflammatory, antioxidant, cardioprotective, antidiabetic, antimalarial, anticancer	143
*Spatholobus suberectus* Dunn (Leguminosae)	China	Aerial parts, wood, stem	Flavonoids, medicarpin, formononetin, isoliquiritigenin	Anti-platelets, anti-inflammatory, anti-bacterial, neuroprotective, anticancer, antioxidant	148, 149
*Radix Astragali* (Fabaceae)	China	Root	Calycosin, formononetin and derivatives, ononin	Hepatoprotective, antidiabetic, analgesics and sedative	150a
*Hedysarum polybotrys* (Leguminosae)	Taiwan, China, Asia	Roots	Flavonoids, triterpenes, coumarins, lignanoids, nitrogen compounds, sterols, carbohydrates and benzofuran	Support immune system, peripheral nerve system, antioxidant, antitumor, anti-aging, anti-diabetic, anti-inflammatory and antiviral activity	195
**(G) Plants having miscellaneous bioactive constituents**					
*Lithospermum erythrorhizon* (Boraginaceae)	Japan, China, Taiwan, Korea	Roots	Shikonin and its derivatives	Macular eruptions, measles, smallpox, eczema, carbuncles and burns, antioxidant, anti-platelet, anti-atherosclerosis, anti-inflammatory	8
*Dioscorea membranacea* (Dioscoreaceae)	Thailand	Rhizomes	Dioscorealide A, dioscorealide B, dioscoreanone, diosgenin	Anticancer, antiallergic, anti-malarial, anti-AIDS	114
*Allium sativum* (Amarylidaceae)	China, Central Asia, Iran, Egypt,	Leaves, bulb,	Organosulfur compound (Diallyl sulfide, diallyl disulfide, allin, allicin etc), saponins, polysaccharides, phenolic compounds	Antioxidant, antidiabetic, antimicrobial, cardiovascular protective, anti-inflammatory, anticancer, anti-obesity, digestive system protective	215
*Emblica officinalis* (Euphorbiaceae)	India, China	Fruit	Vitamin C, alkaloids, phenolic compounds and tannins, minerals, amino acids, proteins	Antidiabetic, antioxidant, anti-cancer, hepatoprotective, hypolipidemic, cardioprotective, antimicrobial	220
*Sorghum bicolor* (Poaceae)	Africa, Asia, Maxico, Nigeria, USA	Grains, whole plant, leaves	Nutrients	Food, liquor, antioxidant, improves immunity	239
*Pentalinon andrieuxii* (Apocynaceae)	West India, America, Mexico, Florida	Roots, leaves	Pentalinonsterol	Immunity, antileishmanial activity against, antiparasitic activity,	240

## Traditional Chinese Medicines (TCM) for Prevention and Treatment of COVID-19

Traditional Chinese Medicine (TCM) is an ancient practice of therapy involving use of various forms of herbal medicines, exercise, acupuncture, dietary therapy and massage. It has been proven to be an effective approach to treat various health problems including viral infections. Recently, some reports were published regarding the effectiveness of the TCM in the prevention and treatment of COVID-19. According to the reports, TCM has been identified to be much effective in the treatment and prevention of COVID-19 and had played vital role in controlling the spread of the disease in China (241–243). Use of decoction based on the TCM principles among the medical professionals prevented the iatrogenic infection of the virus. TCM exhibited excellent curative potential; mainly responsible for early control over the spread of new viral infection and thought to be main reason for faster cure rate and discharge from the hospitals in China in comparison to other countries (1, 241, 244, 245). Treatment of COVID-19 based on TCM has also achieved good results as in one of the study (246), it was reported that out of 1016 diagnosed COVID-19 patients in Hunan province of China, 981 were treated with TCM and 95.76% (779 patients) of them were discharged. Moreover, out of the 233 hospitalized patients, all were cured using TCM with 100% recovery rate (246). In another study, 51 out of 52 COVID-19 patients receiving TCM treatment, showed either improvement or were cured and discharged, with one death (247). Decoction prepared from a mixture of Chinese herbs is the most popular therapy of COVID-19 in TCM and the herbs including *Armeniacae Semen Amarum, Gypsum fibrosum, Glycyrrzhiae Radix et Rhizoma* and *Ephedra herba* were the most commonly mentioned in Chinese guidelines (241, 248).

At molecular level, TCM products were reported to exhibit antiviral property either by directly inhibiting the viral particles by heat clearing and detoxification or indirectly through regulation of body’s immune response. In this regard, *Gentiana, Astragalus, Acanthopaax senticosus* and *Salvia* were found to induce Ig and IFN and produce antiviral activity (241). Chinese Multi herbal formula, Qing Fei Pai Du Tang (QFPDT) optimized for COVID-19 was recommended by government bodies contains quercetin, kaempferol, luteoline, isorrine and naringin as the first five active ingredients. QFPDT was found to act by modulation of body’s immune response, inhibition of inflammatory responses, protection of nerve and lung functions through regulation of MAPK1, MAPK3, MAPK8, IL-6, STA T1 and RELA and signaling pathways TNF and NF-kB (98, 249).

## Future Perspectives

Dysfunction of immune system has been evidently implicated with the development of a number of diseases and modulating and maintaining an efficient immune system has proven to be a justified approach in the treatment of associated diseases. However, there are certain limitations to this approach owing to the complexity of immune system as well as the unavailability of expedient analytical tools to measure the immune functions. Future research on NK cells, cytokine receptors and markers and blood-borne particles are warranted. Despite these limitations, certain medicinal plants have been tested to offer novel avenues for the immune system modulation either alone or in combination and the results are very encouraging. In comparison to other immunity-targeted therapies such as the administration of monoclonal antibodies, plants and their derived products have shown important advantages including abundance and lesser adverse effects.

Nevertheless, these medicinal plants have to undergo standardization assessing both quality of the product and the methodology of research. Another scope of future research on medicinal plants is the chemical modification of active ingredients directed to enhance its efficacy and pharmacokinetic properties. For example, curcumin is not effective alone clinically owing to its absorption and metabolism issue, but chemically modified curcumin (demethoxycurcumin and bis-demoethoxycurcumin) have shown better druggability properties and are under clinical trials. Many of the active constituents of these medicinal plants face the pharmacokinetics related problems and suffer from poor absorption and rapid metabolism leading to poor bioavailability. Several alternate strategies are being adopted and researches are being focused on the development of nanotechnology-based formulations, changing the way of administration and changing the drug delivery technique.

Another promising approach is to test the combination of active constituents obtained from these medicinal plants which could achieve targeted therapy and have synergistic effects with reduced toxicities. Multitarget drugs can also be designed by linking the bioactive molecules by means of suitable spacers and fusion or amalgamation of the pharmacophoric elements which are essential for the activity to get the hybridized molecule. The mechanisms by which these medicinal plants and their bioactive molecules demonstrate immunomodulation are complex and involve multiple signal transduction pathways. Most of these products show extensive and multiple pharmacological effects and their targets and molecular mechanisms are not fully elucidated and understood. Most of the reported biological activities of these medicinal plants are based on cell based *in vitro* and animal based *in vivo* studies, but high level clinical studies are still required to establish their efficacies and toxicities in humans. It is therefore expected that with the advancements in the future molecular level studies, these medicinal plants and their derived products would demonstrate promising breakthroughs and constitute one of the important approaches for immunotherapy.

## Conclusions

Respiratory diseases caused by viral infections including influenza and common cold are globally widespread apart from the repetitive viral outbreaks from SARS-CoV, MERS-CoV and SARS-CoV-2. Direct treatment of such viral infections by modern medicines are not very efficient, and although vaccination is considered to be effective in preventing viral infections, the incidence of acute respiratory infections could be reduced only to some extent. Consequently, alternative treatments for viral infections are necessary, and in this regard, the stimulation of the body’s immune defense system could be an efficient method of preventing these infections. A wide range of plant extracts, herbal products, and pure plant constituents have shown potential immunomodulation effects and used either as prophylactic or therapeutic agents for infectious diseases. Apart from infectious diseases, plant-based immunomodulators can help treat several other immunologic and inflammatory illnesses including cancer, rheumatoid arthritis, plaque psoriasis, Crohn’s disease, etc. These plant products and their bioactive molecules modulate immune responses through the stimulation and modification of lymphocytes, macrophages, and cytokine production. Many plant extracts contain a number of active principles, including polysaccharides, terpenoids, alkaloids, flavonoids, glycosides, and essential oils, which have tremendous capability to maintain and/or stimulate the immune system primarily through the modulation of nonspecific immune responses. However, these plant extracts may contain one or more of the active constituents and their relative contribution and mechanism in the generation of immunomodulation activity are difficult to understand. Isolated pure plant constituents may prove to be specific and effective immunomodulators capable of counteracting the high cost and adverse effects of modern medication. Nevertheless, the drawbacks associated with the application of plant products include the development of standard testing and quality control protocols need to be properly addressed. The present review highlights the importance of medicinal plants containing immunomodulating components of different chemistries with possible utilization in controlling the viral infections. This might stimulate the efforts of researchers towards developing alternative plant-based treatments for infectious diseases.

## Author Contributions

HA and MA developed the concept of the manuscript. Literature survey was conducted by SM, HM and MT. Methodology and data curation were done by SS and WA. SS, SJ and AK wrote the draft manuscript, while writing-review and editing was performed by AN, AM and MA. HA supervised the work. All authors contributed to the article and approved the submitted version.

## Conflict of Interest

The authors declare that the research was conducted in the absence of any commercial or financial relationships that could be construed as a potential conflict of interest.
